# Home-based screen time behaviors amongst youth and their parents: familial typologies and their modifiable correlates

**DOI:** 10.1186/s12889-020-09581-w

**Published:** 2020-10-01

**Authors:** Lauren Arundell, Kate Parker, Anna Timperio, Jo Salmon, Jenny Veitch

**Affiliations:** Institute for Physical Activity and Nutrition (IPAN), School of Exercise and Nutrition Sciences, Deakin University, Geelong, 221 Burwood Highway, Burnwood Victoria, Australia

**Keywords:** Screen time, Sedentary behavior, Children, Families, Typologies, Correlates

## Abstract

**Background:**

Excessive screen time behaviors performed by children and parents at home is a major public health concern. Identifying whether child and parent screen time behaviors cluster and understanding correlates of these familial clusters can help inform interventions for the whole family. This study characterized familial typologies of screen time behaviors and identified key modifiable correlates of these typologies.

**Methods:**

Parents participating in the cross-sectional Sitting in the Home (SIT) study reported the duration (mins/day) they and their child (aged 11.2 ± 2.62 years) spent in six screen time behaviors at home (computer/laptop for home/work, computer/laptop for leisure, TV/videos/DVDs, tablet/smart phone for home/work, tablet/smart phone for leisure, and electronic games) and completed items related to 21 potential correlates framed by an adapted Social Cognitive Theory, Family Perspective. Latent Class Analysis was used to identify typologies based on parent and child data for the six behaviors. Multinomial logistic regression analysis assessed the relative risk of typology membership for each potential correlate, adjusting for child and parent age and sex.

**Results:**

The sample comprised 542 parent-child dyads (parents: 40.7 ± 6.3 yrs., 94% female; children: 11.2 ± 2.6 yrs., 46% female). Three typologies were identified: 1) high computer/moderate TV (*n* = 197); 2) high TV/tablet/smartphone, low computer (*n* = 135); and 3) low-screen users (*n* = 210). ‘Low-screen users’ spent the least amount of time in all screen time behaviors (assigned as reference category). Greater child preference for screen time behaviors, parental support for screen time behaviors and frequency of homework requiring a tablet/laptop were associated with higher odds of being in the ‘high computer/moderate TV’ typology. The odds of being in the ‘high TV/tablet/smartphone, low computer’ typology were greater amongst children with a higher preference for screen time behaviors, and lower among more active parents.

**Conclusions:**

Three familial typologies of screen time behaviors were identified. The findings highlight that screen time in the home can be influenced by the home environment, parental behaviours and role modelling, child preferences as well as school policies. Findings can inform the development of family screen time interventions, however more research exploring the influence of factors outside of the home is warranted.

## Background

The increasing prevalence of sedentary behavior is recognized as a global public health concern [1]. Sedentary behaviors are any sitting, reclining or lying waking behavior requiring an energy expenditure of ≤1.5 metabolic equivalents (METs) [2], many of which are screen-based. Screen time behaviors, such as television viewing (TV), digital tablet, internet and social media use, constitute the majority of children’s and parents’ recreational time [3, 4], and increases their risk of many physical and psychosocial health conditions. For example, compared to children who spend less time using screens, high screen users (i.e., > 2-h/day) are more than twice as likely to exhibit cardio-metabolic risk factors [5, 6], have elevated depression and anxiety symptoms [7], lower academic achievement [8], lower social connectedness [9] and poorer family functioning [10]. Similar negative health outcomes of excessive screen time have also been documented among adults [11, 12]. Although there are currently no Australian screen time recommendations for adults, guidelines for children and adolescents (5–17 years) recommend spending less than 2-h using electronic media per day [13]. However, almost 70% of Australian children do not meet this guideline, with similarly low adherence observed in the United States, Canada and New Zealand [14, 15].

The family and home environment has been identified as a key but under-utilized setting for interventions to reduce children’s screen-based sedentary behaviors [14, 15]. Parents have an influential role on children’s screen time behaviors through role modelling, co-participation and beliefs [16–18], as well as establishing and controlling the home social and physical environment [17], which can either promote or restrict their own and their child’s participation in screen time. Due to the shared home environment, screen time behaviors of parents and their children may cluster together resulting in distinct parent-child dyads. Previous studies have explored correlations between child and parent TV viewing (i.e., co-occurrence) [19] and the agreement between clusters of child screen time and clusters of the mother’s sitting time (i.e., concordance) [20]. Niermann and colleagues [21] explored clustering of familial (mother/father and child) physical activity, total screen time, and dietary intake and identified three clusters defined by parents and children performing similarly high, similarly low, or differing amounts of screen time [21]. However, screen time behaviors in these studies were limited to time watching TV, computer or internet, and computer games, without consideration of the ‘new’ screen time behaviors, such as smartphones and digital tablets, which are readily available and commonly used by both parents and children [4, 22]. To our knowledge, no studies have identified familial (child-parent) typologies of multiple screen time behaviors performed at home. Identification of parent-child typologies of screen time behaviors and their characteristics can guide the development of interventions targeting population sub-groups most in need.

Furthermore, to effectively reduce screen time amongst children and parents in the home environment, it is important to identify the modifiable correlates associated with typology membership. While there is a large body of evidence pertaining to correlates of parents’ and children’s sedentary behaviors separately [23–26], little research has explored influences on clustering of screen-based behaviors (distinct typologies of behavior) among parent-child dyads, particularly in the home setting [27]. Therefore, the aim of this study was to identify and characterize familial typologies of screen time behaviors and examine the modifiable correlates of typology membership.

## Methods

This study used data collected from the Sitting In The home (SIT) Study, a cross-sectional study of parents of 8–16 year old children in Victoria, Australia conducted from October 2017 to February 2018. Methods for the SIT study have been described elsewhere [4]. Briefly, parents of a child aged 8–16 years living in Australia were recruited through social media (e.g., Facebook) and asked to complete an online survey describing their own and their child’s (aged 8–15 years with the next birthday) sedentary behaviors in the home and requested responses to items related to a range of potential correlates. A total of 1925 parents clicked on the study link, 1587 completed the screening questions to start the survey questions, and a total of 553 parents (29%) completed the survey.

The SIT study received ethical approval from the Deakin University Human Research Ethics Committee (HEAG-H 123_2017). Informed consent from participants was obtained at the start of the survey. This manuscript conforms to the STROBE checklist for the reporting of cross-sectional studies (Additional Table 1).

**Fig. 1:**
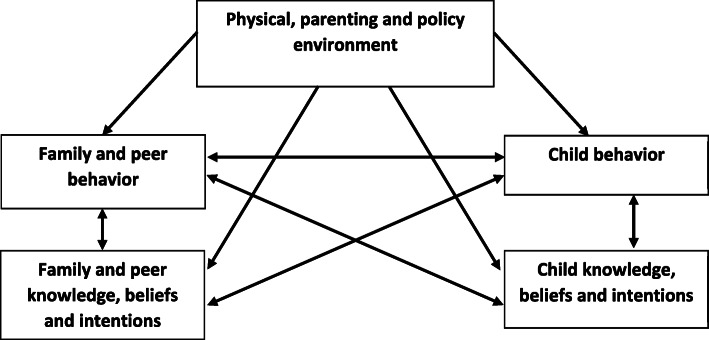
Framework adapted from the Social Cognitive Theory, Family Perspective

This study and survey is based upon an expanded social cognitive theory (SCT). The SCT posits that the individual, their behavior and the environment are in constant, bi-directional reciprocating interaction with one another [28]. The ‘Family Perspective’ expands the SCT to include the behavior and cognition of two or more people as influences on behavior [29]. The current study further extends this framework by distinguishing, for example, physical environment influences (e.g., availability and monitoring of screen devices), parenting environment influences (e.g., parenting strategies, support and rules) and policy environment influences (e.g., school’s requirement of homework using screens). It incorporates cognition within a wider construct of *knowledge, beliefs and intentions*. It also extends the focus from solely exploring parental influences but also includes the influence of family and peers (e.g., sibling and peer role modelling and behavior preference). Lastly, it acknowledges the bi-directional relationship between parent-child behavior and knowledge, beliefs and intentions, which was previously overlooked. The framework used in the current study is shown in Fig. 1.

### Survey measures

#### Demographics

Participants were asked to indicate their own and their child’s sex and age.

#### Screen time behaviors

Parents reported the time (hours and minutes) they and their child spent in six screen time behaviors while sitting on an average week and an average weekend day during school term: (e.g. “*how many minutes does your child spend doing the following activities while sitting at home*”) using the computer or laptop for homework/work; using the computer or laptop for leisure; watching TV/videos/DVDs; using a tablet or smart phone for homework/work; using a tablet or smart phone for leisure; and playing electronic games. After converting hours to minutes, the average duration per day of each behavior was computed ((average weekday × 5) + (average weekend day × 2)/7). To determine test-retest reliability, a subset (*n* = 119) of the current sample completed an identical survey 7 days later. These items were adapted from previous survey items assessing physical activity, sedentary behaviour and screen time [30, 31]. Reliability was determined via intraclass correlation coefficients (ICC) which were considered moderate when between 0.5 and 0.74 and high when above 0.75 [32]. All behaviors were considered at least moderately reliable (ICC 0.65–0.91), except for tablet/smartphone use (ICC 0.45) and games console use (ICC 0.42) which were slightly below this threshold.

#### Potential modifiable correlates

Participants reported on 21 potential modifiable correlates selected based on previous reviews of the correlates of screen time conducted by the authors [3] and others [33]. The survey items were adapted from previous research examining factors from the family and home environment that influence children’s physical activity, sedentary behaviour and screen time [31, 34, 35] or newly developed. The correlates are outlined within the framework shown in Fig. 1. These are described in brief below with a more detailed variable description, coding nomenclature, and data management provided in Additional Table 2. Where required, scale internal reliability was determined via Cronbach’s α.

### Environment

#### Physical environment

Parents reported the number of: screen-based devices, working TVs and electronic games consoles in the home, and the number of screen-based devices, working TVs and electronic games consoles in their child’s bedroom.

#### Parenting environment

Parents reported the existence of rules for TV and electronics use, frequency of emotional support for screen time behaviors (e.g., encourage child to sit quietly and watch TV at home), the frequency they use screen behaviors to keep their child occupied, and parent discouragement of screen time behaviors.

#### Policy environment

Parents reported how often their child’s homework requires a tablet/laptop.

### Family and peer behaviors

Parents reported the frequency they met the physical activity recommendations, and the frequency that their child participated in screen time behaviors with siblings, a parent/guardian and peers.

### Family and peer knowledge, beliefs and intentions

Parents reported their concerns about screen time behaviors (e.g.; I am concerned about what my child may be exposed to when using electronic media).

### Child behavior

Parents reported their child’s average sleep duration (hours/minutes) and the frequency they met the physical activity recommendations (days/week performed at least 60 min of moderate- to vigorous intensity physical activity).

### Child knowledge, beliefs and intentions

Parents reported their child’s preference for screen-based behaviors and whether they considered their child ‘addicted’ to electronic media.

#### Data analysis

Each of the 12 screen-based variables described above (six for children and for parents) were dichotomized based on their mean to categorize high versus low screen use. This was due to the large number of participants who reported zero minutes of some behaviors. Latent class analysis (LCA) was conducted based on these 12 dichotomized variables using MPlus statistical software. LCA handles missing data using maximum likelihood estimations and therefore could be conducted on the 542 participants who had reported both the child and parent data for at least one of these screen based behaviors (missing data for the 12 variables ranged from 8 to 29%). The optimal class solution was determined by comparing two- through five-class models based on key statistical indicators, including Akaike Information Criteria (AIC) [36], Bayesian Information Criteria (BAI) [37], Lo-Mendell-Rubin likelihood ratio test [38], Entropy [39] and class sizes. The three-class solution was the only solution where Lo-Mendell-Rubin likelihood ratio test were significant while maintaining a low AIC, BIC and high Entropy, and relatively balanced sample sizes.

The optimal class solution, referred to as familial typologies of screen time behaviors, was imported into STATA (version 15) for all further analyses. One-way ANOVAs were used to compare typologies based on parent or child age and each of the 21 potential correlates. Chi-square tests were used to determine differences with respect to the sex of the parent and child. Significance was set at *p* < 0.05. Multinomial logistic regression (MNLR) analysis determined the relative risk (RR) of being classified in each typology based on each of the 21 potential modifiable correlates, with the most optimal typology (lowest screen time behavior) used as the reference category. Each potential correlate was first entered into the model individually, adjusting for the age of the child and parent, and sex of the child (significant demographic characteristics). Those that were significant (*p* < 0.05) were included in the fully adjusted model. Collinearity of independent variables was also checked to ensure it was not impacting the findings.

## Results

### Sample

The final sample included 542 parent-child dyads. Parents were 40.7 ± 6.3 years of age and 93.5% were female. Children were 11.2 ± 2.6 years of age and 46% were female.

### Familial typologies of screen time behaviors

**Fig. 2:**
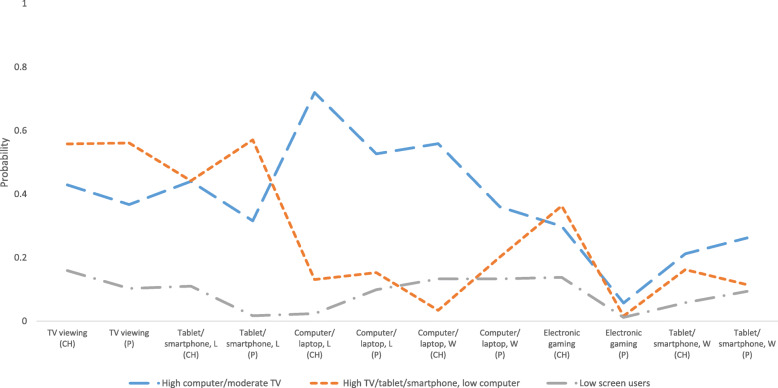
Item-response probability plot for familial typologies of screen time behaviors. Abbreviations: CH = child; P = parent; L = for leisure; W = for homework/work

Each of the three classes (typologies) were labelled based on the behaviors which were most prevalent and distinguished the typology from the others. Typology 1 was labelled ‘high computer/moderate TV’ (*n* = 197); typology 2 ‘high TV/tablet/smartphone, low computer’ (*n* = 135); and typology 3 ‘low-screen users’ (*n* = 210). Figure 2 shows the item-response probability plot which provides a visual representation of the relative contribution of each screen time behavior towards typology composition.

### Characteristics of familial screen typologies

The ‘high TV/tablet/smartphone, low computer’ typology had the lowest proportion of girls compared to the other typologies, *p* = 0.046 (Table 1). Parents and children in the ‘high computer, moderate TV’ typology were significantly older than both other typologies (*p* < 0.01 for both). Parents and children in the ‘low-screen users’ typology spent less time participating in all six screen time behaviors than those participants in the ‘high computer/moderate TV’ typology and significantly less time TV viewing and using a tablet/smartphone for leisure. Children in the ‘low-screen users’ typology spent less time using a tablet/smartphone for homework, than those participants in the ‘high TV/tablet/smartphone, low computer’ typology. Compared to participants in the ‘high computer/moderate TV’ typology, participants in the ‘high TV/tablet/smartphone, low computer’ typology spent significantly more time watching TV, playing electronic games and using a tablet/smartphone use for leisure (parents only), but less time using a computer for homework or leisure, and a tablet/smartphone use for homework (parents only).

**Table 1:** Child and parent demographics and average screen time behaviors (mean minutes/day [±SD]) by screen time behavior typology membership

	Whole sample ***n*** = 542	Low screen users ***n*** = 210	High computer/ moderate TV ***n*** = 197	High TV/tablet/smartphone, low computer ***n*** = 135	***P*** value
	Mean (SD)	Mean (SD)	Mean (SD)	Mean (SD)	
Demographics					
Parent sex (% women)	93.5	93.3	92.9	94.8	*0.915*
Parent age	40.9 (6.16)	40.3 (5.78)^a^	41.9 (6.64)^ab^	39.5 (6.32)^b^	*0.0017*
Child sex (% girls)	46.2	49.0^a^	49.7^b^	36.3^ab^	*0.046*
Child age	11.2 (2.62)	10.4 (2.43)^ab^	12.2 (2.64)^ab^	10.7 (2.36)^b^	*< 0.001*
Screen time behaviors					
*Children*					
TV viewing	67.22 (59.03)	43.68 (41.86)^ac^	71.88 (60.87)^ab^	91.9 (64.39)^bc^	*p < 0.001*
Tablet/smartphone (leisure)	55.32 (72.06)	26.96 (41.98)^ab^	69.08 (72.32)^a^	72.48 (89.61)^b^	*p < 0.001*
Computer (leisure)	45.36 (72.58)	7.17 (13.65)^a^	96.51 (86.25)^ab^	14.03 (38.16)^b^	*p < 0.001*
Computer (homework)	25.84 (59.17)	9.54 (19.75)^a^	54.14 (84.35)^ab^	6.27 (22.75)^b^	*p < 0.001*
Electronic gaming	22.67 (53.71)	7.06 (21.43)^ac^	24.3 (52.83)^ab^	41.74 (75.56)^bc^	*p < 0.001*
Tablet/smartphone (homework)	6.3 (19.54)	1.54 (7.62)^a^	11.27 (28.11)^a^	5.86 (13.77)	*p < 0.001*
*Parents*					
TV viewing	61.38 (62.90)	32.09 (34.56)^ac^	68.15 (71.36)^ab^	88.04 (63.25)^bc^	*p < 0.001*
Tablet/smartphone (leisure)	79.8 (102.05)	26.2 (25.95)^ac^	86.11 (107.05)^ab^	134.46 (119.01)^bc^	*p < 0.001*
Computer (leisure)	34.71 (62.31)	16.26 (41.49)^a^	61.73 (76.46)^ab^	18.95 (45.67)^b^	*p < 0.001*
Computer (homework)	45.52 (89.45)	29.03 (59.02)^a^	72.26 (119.16)^ab^	27.78 (56.4)^b^	*p < 0.001*
Electronic gaming	1.53 (12.46)	0.29 (3.16)^a^	3.79 (20.04)^ab^	0.07 (0.76)^b^	*p = 0.01*
Tablet/smartphone (homework)	11.06 (28.99)	6.4 (18.25)^a^	18.16 (38.28)^ab^	7.04 (22.53)^b^	*p < 0.001*

Abbreviations: *SD* Standard deviation, *CH* Child, *P* Parent. Note: Typologies with the same superscript letter (i.e.^a,b^ or ^c^) are significantly different from each other

Table 2 shows mean scores for each of the potential correlates overall and according to familial screen time behavior typology. There were 11 significant differences in characteristics between the typologies. Compared to ‘high computer/moderate TV’ and ‘high TV/tablet/smartphone, low computer’, ‘low screen users’ had the most sleep, met the physical activity recommendations more frequently. They also had the lowest screen-based sedentary behavior preference score, more restrictive rules around the use of TV and electronics, used sedentary behaviors to keep the child occupied least frequently, and the child’s bedroom had the lowest number of screen devices. Compared to ‘high computer/moderate TV’, children defined as ‘low screen users’ participated in sedentary behaviors with peers less frequently, and the home environment had fewer screen-based devices. Parents in the ‘low screen users’ typology also met the physical activity recommendations more often than those in the ‘high TV/tablet/smartphone, low computer’ typology. The one anomaly was for the frequency with which children’s homework required them to use a tablet or laptop. Children in the ‘low screen users’ typology were required to use a tablet or laptop for homework more often than children in the ‘high TV/tablet/smartphone, low computer’, but less often than the ‘high computer/moderate TV’ group.

**Table 2:** Mean (SD) characteristics by screen time behavior typology membership

	Whole sample	***Low screen users***	***High computer/moderate TV***	***High TV/tablet/smartphone, low computer***	***P*** value
	Mean (SD)	Mean (SD)	Mean (SD)	Mean (SD)	
Environment					
*Physical Environment*					
Screen-based devices in the home (n)	4.60 (1.10)	4.43 (1.11)^a^	4.82 (1.10)^a^	4.54 (1.04)	**0.003**
Working TVs in house (n)	2.44 (2.46)	2.46 (3.64)	2.38 (1.26)	2.49 (1.15)	0.93
E-games consoles house (n)	1.50 (2.99)	1.39 (4.37)	1.58 (1.56)	1.55 (1.61)	0.80
Screen-based devices in child’s bedroom (n)	1.58 (1.41)	1.23 (1.36)^a^	1.97 (1.42)^ab^	1.55 (1.34)^b^	**< 0.001**
Working TVs in child’s bedroom (n)	0.46 (2.88)	0.60 (4.62)	0.38 (0.54)	0.35 (0.48)	0.72
E-games consoles in child’s bedroom (n)	0.45 (3.86)	0.62 (6.19)	0.36 (0.63)	0.62 (0.57)	0.77
*Parenting Environment*					
Rules for TV use	4.36 (3.95)	5.25 (3.65)^ab^	3.66 (4.20)^a^	3.99 (3.80)^b^	**< 0.001**
Rules for electronics use	5.09 (5.37)	6.44 (5.04)^b^	3.61 (5.34)^ab^	5.15 (5.39)^a^	**< 0.001**
Emotional support for screen time behaviors	5.64 (6.36)	5.12 (5.75)	6.19 (7.30)	5.68 (5.77)	0.32
Use of screen behaviors to keep child occupied	−2.36 (2.10)	−2.73 (2.11)^ab^	−2.17 (2.08)^a^	− 2.07 (2.04)^b^	**0.004**
Parent discouragement of screen time behaviors	9.89 (7.21)	10.08 (7.66)	9.67 (7.06)	9.90 (6.71)	0.87
*Policy Environment*					
Child’s homework requires a tablet/laptop	1.69 (1.04)	1.51 (1.03)^a^	2.02 (0.99)^ab^	1.50 (0.99)^b^	**< 0.001**
Family and peer behavior					
Parental physical activity (days/week meet recs)	2.77 (2.10)	3.00 (2.11)^a^	2.77 (2.17)	2.39 (1.94)^a^	**0.032**
Screen time behaviors co-participation: Siblings	2.82 (2.58)	2.67 (2.49)	2.78 (2.51)	3.13 (2.79)	0.25
Screen time behaviors co-participation: Parent/ guardian	2.35 (2.30)	2.28 (2.27)	2.28 (2.31)	2.56 (2.35)	0.48
Screen time behaviors co-participation: Peers	1.24 (1.74)	0.97 (1.52)^a^	1.54 (2.02)^a^	1.25 (1.54)	**0.004**
Family and peer knowledge, beliefs and intentions					
Parent concerns about screen time behaviors	1.40 (1.64)	1.43 (1.76)	1.27 (1.61)	1.56 (1.50)	0.27
Child behavior					
Child’s sleep (minutes/night)	548.85 (70.73)	564.62 (70.15)^b^	528.64 (69.92)^ab^	553.59 (66.08)^a^	**< 0.001**
Child’s physical activity (days/week meet recs)	3.45 (2.15)	3.73 (2.09)^a^	3.21 (2.20)^a^	3.38 (2.11)	**0.045**
Child knowledge, beliefs and intentions					
Child’s preference for screen time behaviours	−0.00 (1.25)	−0.38 (1.18)^ab^	0.28 (1.26)^a^	0.17 (1.21)^b^	**< 0.001**
Child’s addiction to electronic media (parental perceived)	2.92 (1.28)	2.56 (1.24)^ab^	3.24 (1.28)^a^	3.04 (1.21)^b^	**< 0.001**

Note: Typologies with the same superscript letter (i.e.^ a,b^ or ^c^) are significantly different from each other; significant differences are bolded. Abbreviations: *PA* Physical activity, *SB* Sedentary behavior

### Modifiable correlates associated with familial screen time behaviors typologies

Four correlates were associated with familial screen time behavior typologies based on the adjusted multinomial logistic regression (Table 3), including one from each of the parenting/family environment, the policy environment, family and peer behavior and child knowledge, beliefs and intentions areas. For each unit increase in parental encouragement and support of the child’s screen time behavior, parent-child dyads were 7% more likely to be classed as ‘high computer/moderate TV’ compared to ‘low screen users’. For each unit increase in the frequency that the child’s homework required a tablet or computer, there was a 43% greater odds of parent-child dyads being classed as ‘high computer/moderate TV’ compared to ‘low screen users’. For each additional day that the parent engaged in at least 30-min of physical activity, parent-child dyads were 15% less likely to be defined as ‘high TV/tablet/smartphone, low computer’ compared to ‘low screen users’. For each unit increase in child preference for screen time behavior, parent-child dyads were 56 and 35% more likely to be classified within the ‘high computer/moderate TV’ and ‘high TV/tablet/smartphone, low computer’ typologies, respectively, compared to the ‘low screen users’.

**Table 3:** Relative risk of screen time behavior typology membership according to potential correlates from the adapted Social Cognitive Theory, Family Perspective

	Unadjusted MNLR results^a^			Adjusted MNLR results^b^		
	*Low screen users*	*High computer/ moderate TV*	*High TV/tablet/ smartphone, low computer*	*Low screen users*	*High computer/ moderate TV*	*High TV/tablet/ smartphone, low computer*
	Ref	RR (95% CI)	RR (95% CI)	Ref	RR (95% CI)	RR (95% CI)
Environment						
*Physical Environment*						
Screen-based devices in the home (n)	1.00	**1.42 (1.15–1.76)**	1.08 (0.88–1.33)	1.00	1.27 (0.93–1.72)	0.93 (0.69–1.24)
Working TVs in house (n)	1.00	0.97 (0.88–1.06)	0.99 (0.91–1.08)	–	–	–
e-games consoles in house (n)	1.00	1.00 (0.93–1.08)	1.00 (0.93–1.09)	–	–	–
Screen-based devices in child’s bedroom (n)	1.00	**1.25 (1.04–1.51)**	1.13 (0.93–1.36)	1.00	0.96 (0.73–1.25)	0.97 (0.75–1.26)
Working TVs in child’s bedroom (n)	1.00	0.95 (0.85–1.07)	0.95 (0.83–1.10)	–	–	–
e-games consoles in child’s bedroom (n)	1.00	0.97 (0.90–1.04)	0.97 (0.87–1.07)	–	–	–
*Parenting Environment*						
Rules for TV use	1.00	**1.06 (1.01–1.13)**	**1.08 (1.02–1.15)**	1.00	1.03 (0.92–1.17)	0.93 (0.83–1.05)
Rules for electronics use	1.00	**1.06 (1.02–1.11)**	1.04 (0.99–1.09)	1.00	0.91 (0.82–1.00)	0.98 (0.89–1.08)
Emotional support for screen time behaviors	1.00	**1.05 (1.01–1.10)**	1.02 (0.98–1.07)	1.00	**1.07 (1.01–1.14)**	1.06 (0.99–1.12)
Use of STBs to keep child occupied	1.00	**1.13 (1.02–1.26)**	**1.15 (1.03–1.28)**	1.00	0.94 (0.78–1.12)	0.91 (0.76–1.09)
Parental discouragement of screen time behaviors	1.00	1.00 (0.97–1.03)	0.99 (0.96–1.03)	–	–	–
*Policy Environment*						
Child’s homework requires a tablet/laptop	1.00	**1.27 (1.02–1.59)**	0.95 (0.75–1.19)	1.00	**1.43 (1.03–1.99)**	0.94 (0.69–1.29)
Family and peer behavior						
Parent physical activity (days/week meet PA recs.)	1.00	0.91 (0.82–1.00)	**0.86 (0.77–0.95)**	1.00	0.88 (0.76–1.02)	**0.85 (0.74–0.99)**
Sibling screen co-participation	1.00	1.08 (0.99–1.17)	1.07 (0.98–1.17)	–	–	–
Parent/guardian screen co-participation	1.00	1.05 (0.96–1.15)	1.05 (0.96–1.16)	–	–	–
Peer screen co-participation	1.00	**1.18 (1.04–1.34)**	1.10 (0.96–1.27)	1.00	1.05 (0.88–1.26)	1.00 (0.83–1.22)
Family and peer knowledge, beliefs and intentions						
Parent concerns about screen time behaviors	1.00	0.99 (0.87–1.13)	1.05 (0.92–1.20)	–	–	–
Child behavior						
Child’s sleep (minutes/night)	1.00	**0.996 (0.993–0.999)**	0.998 (0.994–1.001)	1.00	0.99 (0.99–1.00)	0.99 (0.99–1.00)
Child’s physical activity (days/week meet recs)	1.00	0.93 (0.84–1.02)	0.91 (0.82–1.01)	–	–	–
Child knowledge, beliefs and intentions						
Child’s preference for screen time behaviors	1.00	**1.43 (1.21–1.70)**	**1.40 (1.16–1.68)**	1.00	**1.56 (1.16–2.08)**	**1.35 (1.01–1.81)**
Child’s addiction to electronic media (parental perceived)	1.00	**1.44 (1.21–1.70)**	**1.30 (1.09–1.55)**	1.00	1.06 (0.79–1.41)	1.05 (0.79–1.40)

Abbreviations: *MNLR* Multinomial logistic regression; ^a^adjusted for child and parent age and sex; ^b^adjusted for child and parent age and sex, and all other variables significant in the unadjusted analysis; *RR* Relative risk of typology membership, *CI* Confidence interval, *PA* Physical activity, *SB* Sedentary behavior; significant associations are bolded

## Discussion

This study was the first to identify and examine correlates of distinct typologies of multiple screen time behaviors performed by both children and their parents in the home setting. Three distinct typologies of screen time behaviors were classified based on the unique combinations of screen time behaviors performed by the child and parent. The largest group were classified as (relatively) low users, the second largest group performed high levels of computer use for homework and leisure, and the smallest group performed high levels of TV viewing, and tablet/smartphone use for homework and leisure. A diverse range of influences on screen time behaviors were highlighted as the four correlates associated with the familial typologies of screen time were from four different domains of the adapted Social Cognitive Theory, Family Perspective framework. The findings emphasize the importance of the shared home environment, the potential impact of parental role modelling and observational learning, as well as the influence school policy may have on families screen time [40, 41]. The identification of distinct familial typologies, and correlates further highlights that not all families are the same and a ‘one size fits all’ approach to interventions may not provide optimal effects for all.

Child’s preference for screen time behaviors was the strongest correlate of screen time behavior typology, and was the only factor significantly associated with a family being characterized in both ‘high computer/moderate TV’ and ‘high TV/tablet/smartphone, low computer’ typologies. Although parental preference for sedentary behavior was not assessed in the current study it has been shown to be a consistent correlate of health behavior participation amongst adults [42], and future research should also determine if it is associated children’s behavior. The current finding that child preference for screen time behaviors was associated with familial screen time behavior typology membership further builds the rationale for our adaptation of the Social Cognitive Theory, Family Perspective by including the relationship between child knowledge, beliefs and intentions and family and peer behaviors. The bi-directional nature of this relationship wasn’t examined in this study but should be considered in future research that also examines children, families and peer groups.

While the current study focused on screen time behaviors within the home setting, the findings show that these behaviours are influenced by the wider environment including school expectations on the use of screens for homework. This is particularly relevant for the families characterized by high computer for leisure and homework/work and moderate TV viewing. With increasing requirements to use a tablet/computer for homework, families were more likely (43%) to be characterized as ‘high computer/moderate TV’ compared to a ‘low-user’. Interestingly, this typology was characterized by high levels of computer use for both homework/work and leisure time, suggesting that children may continue to use the computer once they have completed their homework. Further, while children are doing their prescribed homework, it is possible that parents use this time to complete their own work tasks which may explain the similar computer use among children and their parents. However, further research is needed to explore this. Another potential explanation is that parents’ within this typology support their child to use screens to enable them to complete their work tasks. The current findings showed that parental emotional support for screen time behaviors was associated with an increased likelihood of being in the typology defined as ‘high computer/moderate TV’ compared to ‘low screen users’. Further, the majority of the measures in the emotional support for sedentary behavior score were specific to encouragement of computer use, which strengthens this potential explanation. While this finding is similar to previous research showing that parental support is an important influence on children’s health behaviors [41, 43], the impact of emotional support for use of other screen types may be important to assess in future studies.

Parents and children in the ‘high computer/moderate TV’ typology were older than the parents and children in the other typologies and their behavior may be reflective of the increased requirement for homework as children become older. Schools may therefore need to enforce policies regarding the frequency that their homework tasks require a tablet/computer, as well as appropriate use of school technologies for recreational purposes. Intervention strategies should include helping families to instill rules and expectations to turn off the tablet/computer once homework is completed and for parents to role model these desired behaviors. The development of software to assist in limiting the use of tablets or computers for non-school purposes may also assist parents and children to reduce excessive leisure time screen use and may be a strategy for future interventions.

The only correlate associated with the likelihood of being in the ‘high TV/tablet/smartphone, low computer’ was parental achievement of the Australian physical activity recommendations (at least 30 min of physical activity per day [13]). Each additional day the parent achieved the guidelines, parent/child dyads were 15% less likely to be within this typology compared to ‘low screen users’. It may also be that parents are role modelling lower engagement with more discretionary screen use (i.e. TV, tablet, smart phone use) [44]. Intervention and public health strategies that encourage parental physical activity may therefore have additional flow on effects for other health behaviors and may help to reduce their child’s screen time.

There were a number of potential correlates examined in this study that were not associated with child and parent screen time behavior typology membership despite being associated with individual screen behavior types in children and in adolescents, respectively. For example, rules and restrictions on children’s screen use were not associated with typology in the current study but have previously been shown to be inversely associated with children’s screen time [45, 46] but not with parents’ screen time [47]. Future research should examine whether parents who place restrictions on their own screen use, spend less time on screens. In addition, no home physical environmental correlates were associated with typology membership which is in contrast to previous research that has shown availability of screen devices to be positively associated with children’s screen time [48, 49]. The lack of association may be due to the limited variability in the number and type of screens in the home (e.g. 99.9% had a TV). Also, the portability of screens and the multitude of activities that can now be performed on the same device (e.g., computing, stream videos, chat etc.) may mean that families do not require as many screens. In addition, there may be other key physical environmental factors associated with participation in individual and ‘new’ screens (e.g., location and proximity of internet access) that were not assessed but should be explored in future research.

A limitation of the current study was the use of parent report of their own and proxy report of their child’s screen time behaviors. Although the items were adapted from reliable and valid items assessing parental report of a limited number of child screen behaviors [30, 31], future research should assess the reliability and validity of this more comprehensive list of screen behaviors. In addition, the survey items did not capture concurrent screen use (e.g., using smartphone while watching TV). The use of dichotomous variables to determine typologies of screen time behaviors may also have reduced the ability to be able to identify differences in behaviors and associated correlates. The recruitment of participants via social media may have resulted in volunteer bias, however data were not collected from non-participants and therefore, this cannot be determined. Strengths include the large sample of parent-child dyads. The inclusion of multiple screen time behaviors, including ‘new’ options such as tablet/smartphone use, also provides better understanding of factors associated with participation in these behaviors which are now commonly performed [4]. This information is key to the development of targeted intervention strategies for children and parents and builds on the importance of the family environment in influencing screen time [50]. Lastly, this study examined the correlates of familial typologies of screen time behaviors across areas of influence from an adapted Social Cognitive Theory, Family Perspective framework which enables multiple levels of influence to be identified. Future research may extend this work but examining differences by age or school levels or amongst different population groups.

## Conclusion

This study identified three unique familial typologies of parent-child screen time behaviors using a variety of ‘new’ and ‘traditional’ screen behaviors performed in the home setting. Within each typology, patterns of screen time behaviors were similar between parents and their children, emphasizing the importance of the home environment in shaping and inhibiting screen-based sedentary behaviors for all family members. Apart from child’s preference for screen time, correlates associated with typology membership differed suggesting that these are distinct intervention target groups, and that the nature of the interventions and the mediators of change that should be targeted, may also differ.

## Supplementary information

**Additional file 1: Table S1.** STROBE Statement—Checklist of items that should be included in reports of cross-sectional studies. This file contains the STROBE checklist for the current manuscript.

**Additional file 2: Table S2.** Parent proxy-reported survey items examining the correlates of children’s and parent’s screen time behavior typologies. This file contains the description of the survey variable description, coding nomenclature, and data management, and where required, scale internal reliability (Cronbach’s α).

## Data Availability

The datasets used and/or analyzed during the current study are available from the corresponding author on reasonable request.

## Abbreviations

CI: Confidence interval
CH: Child
LCA: Latent Class Analysis
L: Leisure
METs: Metabolic equivalents
P: Parent
PA: Physical activity
RR: Relative risk of typology membership
SB: Sedentary behavior
SD: Standard deviation
TV: Television
W: Homework/work
