# Supplementary material for: Home-based screen time behaviors amongst youth and their parents: familial typologies and their modifiable correlates
Source: BMC Public Health. 2020 Oct 1;20:1492. doi: 10.1186/s12889-020-09581-w (PMC7528232; doi:10.1186/s12889-020-09581-w)
Supplement: Supplementary file 2 — Additional file 2: Table S2. Parent proxy-reported survey items examining the correlates of children’s and parent’s screen time behavior typologies. This file contains the description of the survey variable description, coding nomenclature, and data management, and where required, scale internal reliability (Cronbach’s α). [file 12889_2020_9581_MOESM2_ESM.docx]

**Additional Table 2:** Parent proxy-reported survey items examining the correlates of children’s and parent’s screen time behavior typologies.

| **Construct** | **Variable description** | **Response options and variable coding** | **Range** | **Reliability Scale Cronbachs alpha** |
| --- | --- | --- | --- | --- |
| Environment | | | | |
| *Physical Environment* | | | | |
| Screen-based devices in the home (n) | In your home: Pay TV; Internet; Video/DVD player; Nintendo Wii; Smart phone/ iPhone; Digital Tablet | 1 = yes, 0=no | 0-6 |  |
| Working TVs in house (n) | How many workings TVs in home? |  |  |  |
| e-games consoles house (n) | How many e-games consoles in home? |  |  |  |
| Screen-based devices in child’s bedroom (n) | In child’s room/child owns: Pay TV; Internet; Video/DVD player; Nintendo Wii; Smart phone/iPhone; Digital Tablet | 1 = yes, 0=no | 0-6 |  |
| Working TVs in child’s bedroom (n) | How many workings TVs in child’s room? |  |  |  |
| e-games consoles in child’s bedroom (n) | How many e-games consoles in child’s room? |  |  |  |
| *Parenting Environment* | | | | |
| TV rules | My child is allowed to: Watch any television shows he/she chooses; Watch TV when he/she chooses; Watch TV during meal times; Watch TV/use the computer/smart phone/digital before completing his/her homework; Watch TV in his/her bedroom | -2 strongly disagree, -1 disagree, 0=neither agree/disagree, 1=agree, 2=Strongly agree | -10 – +10 | 0.73 |
| Electronics rules | My child is allowed to: Use the smart phone/digital tablet for anything he/she chooses; Play on the computer for anything he/she chooses; Use the smart phone/digital tablet when he/she chooses; Play on the computer when he/she chooses; Use the smart phone/digital tablet in his/her bedroom; Play on the computer/use electronic games (e.g. PSP) in his/her bedroom | -2 strongly disagree, -1 disagree, 0=neither agree/disagree, 1=agree, 2=Strongly agree | -12 – +12 | 0.88 |
| Emotional support for screen-based SB | How often per week do you: Encourage your child to sit quietly and watch TV at home; Encourage your child to sit quietly and use computer/electronic games at home; Encourage your child to sit quietly and use a smart phone/ digital tablet at home; Watch your child play on the computer/electronic games; Praise your child for playing on the computer/electronic games | 0=never, 1.5=1-2 days/wk, 3.5=3-4 days/ wk, 5.5=5-6 days/wk, 7=Everyday, ‘.’=N/A | 0-7 |  |
| Use of TV to keep child occupied | How much do you agree with: If my child used electronic media less, there would be nothing for my child to do; Electronic media keeps my child occupied and out of trouble; I am too busy to be concerned about how much time my child spends sitting | -2 strongly disagree, -1 disagree, 0=neither agree/disagree, 1=agree, 2=Strongly agree | -6 - +6 | 0.6 |
| Parental discouragement of screen-based sedentary behaviours | How often per week do you: Discourage your child from watching too much TV at home; Discourage your child from using the computer/electronic games at home; Discourage your child from using the smart phone/ digital tablet at home | 0=never, 1.5=1-2 days/wk, 3.5=3-4 days/ wk, 5.5=5-6 days/wk, 7=Everyday, ‘.’=N/A | 0-7 |  |
| *Policy Environment* | | | | |
| Homework requires tablet/laptop | My child’s homework requires him/her to use a tablet/laptop | 0 = never; 1=rarely, 2=sometimes, 3=always |  |  |
| Family and peer behaviour | | | | |
| Parental PA | In the last week, how many days did you perform moderate-to vigorous-intensity physical activity for at least 30 minutes |  | 0-7 |  |
| SB co-participation: Sibling | In a typical week, frequency that child sits and watch TV, play video/electronic games, on the computer, or with other electronic devices with siblings | 0=never, 1.5=1-2 days/wk, 3.5=3-4 days/ wk, 5.5=5-6 days/wk, 7=Everyday, ‘.’=N/A | 0-7 |  |
| SB co-participation: Parent/guardian | In a typical week, frequency that child sits and watch TV, play video/electronic games, on the computer, or with other electronic devices with parent/guardian/caregiver | 0=never, 1.5=1-2 days/wk, 3.5=3-4 days /wk, 5.5=5-6 days/wk, 7=Everyday, ‘.’=N/A | 0-7 |  |
| SB co-participation: Peers | In a typical week, frequency that child sits and watch TV, play video/electronic games, on the computer, or with other electronic devices with peers | 0=never, 1.5=1-2 days/wk, 3.5=3-4 days/ wk, 5.5=5-6 days/wk, 7=Everyday, ‘.’=N/A | 0-7 |  |
| Family and peer knowledge, beliefs and intentions | | | | |
| Parental concerns about screen time behaviours | How much do you agree with: Spending too much time on electronic media negatively effects my child's behaviour; I am concerned about what my child may be exposed to when using electronic media | -2 strongly disagree, -1 disagree, 0=neither agree/disagree, 1=agree, 2=Strongly agree | -4 - +4 |  |
| Child behaviour | | | | |
| Child sleep (minutes/night) | On average, how many hours/minutes of sleep does your child have per night? |  |  |  |
| Child physical activity  (Days/week meet recommendations) | In the last week, how many days did your child perform moderate-to vigorous-intensity physical activity for at least 60 minutes? |  |  |  |
| Child knowledge, beliefs and intentions |  |  |  |  |
| Child’s preference for screen-based behaviors | My child prefers to use electronic media than do physical activity or play outside | -2 strongly disagree, -1 disagree, 0=neither agree/disagree, 1=agree, 2=Strongly agree | -2-+2 |  |
| Child’s addiction to electronic media (parental perceived) | My child is ‘addicted’ to electronic media | -2 strongly disagree, -1 disagree, 0=neither agree/disagree, 1=agree, 2=Strongly agree | -2-+2 |  |

Abbreviations: PA=physical activity, SB=sedentary behaviour, rev. coded = reverse coded, freq. = frequency
